# Life satisfaction among active socially engaged community dwelling older adults from Sao Paulo city Brazil

**DOI:** 10.1002/alz70858_101360

**Published:** 2025-12-25

**Authors:** Kauane AM Macedo, Gabriela dos Santos, Tiago Nascimento Ordonez, Sabrina Aparecida Da Silva, Laydiane Alves Costa, Ana Paula Bagli Moreira, Maria Antônia Antunes Fernandes, Diana Dos Santos Bacelar, Gabriela Cristina Siqueira, Patrícia Prata Lessa, Neide Pereira Cardoso, Luiz Carlos Moraes, Sonia Maria Dozzi Brucki, Thais Bento Lima Silva

**Affiliations:** ^1^ Universidade de São Paulo, São Paulo, São Paulo, Brazil; ^2^ Gerontology of the School of Arts, Science and Humanities of the University of São Paulo, São Paulo, São Paulo, Brazil; ^3^ USP, São Paulo, São Paulo, Brazil; ^4^ Supera Institute of Education, São José dos Campos, São Paulo, Brazil; ^5^ University of São Paulo School of Medicine, São Paulo, São Paulo, Brazil; ^6^ University of São Paulo, São Paulo, São Paulo, Brazil

## Abstract

**Background:**

In Brazil and globally, studies report a high prevalence of depressive and anxious symptoms among the older population, negatively impacting mental health and well‐being in late life, including limitations in sociability and social interactions.

**Method:**

A cross‐sectional study was conducted involving the application of the Brazilian telephone version of the Mini‐Mental State Examination (Braztel‐MMSE), the Geriatric Depression Scale (GDS‐15), and Geriatric Anxiety Scale (GAI). A question probing the presence or absence of changes in mood and/or anxiety was also applied. This was a telephone‐based study.

**Result:**

The sample comprised 576 older adults with a mean age of 67.73±5.65.years, predominantly female, married, and with mean education of 15.17±3.75 years. Mean score on the Braztel‐MMSE was 20.27 ±1.48 out of a total of score of 22. Mean score on the GDS‐15 was 2.55±2.32. Mean total score on the GAI was 3.62±4.10, and 70.5% reported changes in mood and anxiety due to the prevailing challenges regarding safety and social inequalities in the community. A significant correlation between total scores on the Braztel‐MMSE and GDS‐15 was evident only in the sample that reported changes in mood and anxiety during the pandemic (rho=‐0.14; *p* = 0.004). Results suggest an association between depressive symptoms and cognitive performance in the older adults assessed. However, this association was more evident when individuals were analyzed separately for presence or absence of changes in mood and/or anxiety.

**Conclusion:**

The older adults at most risk of developing psychological disorders are those living in circumstances of social vulnerability, that have chronic health conditions, and that are more removed from factors considered protective, such as higher working wage, greater educational level, lower use of medications, and fewer self‐reported diseases. These findings can help inform planning of services and public policies for older individuals in the pandemic era, particularly in developing low‐to‐middle income countries experiencing concomitant social inequalities, climate change and increase in chronic diseases.

